# Hybrid ZnO NR/graphene structures as advanced optoelectronic devices with high transmittance

**DOI:** 10.1186/1556-276X-8-350

**Published:** 2013-08-10

**Authors:** Ren-Jei Chung, Zih-Cian Lin, Po-Kang Yang, Kun-Yu Lai, Shou-Feng Jen, Po-Wen Chiu

**Affiliations:** 1 Department of Chemical Engineering and Biotechnology, National Taipei University of Technology (Taipei Tech), Taipei 10608, Taiwan, Republic of China; 2 Institute of Photonics and Optoelectronics, Department of Electrical Engineering, National Taiwan University, Taipei 10617, Taiwan, Republic of China; 3 Department of Optics and Photonics, National Central University, Jhongli City, Taoyuan County 32001, Taiwan, Republic of China; 4 Department of Electrical Engineering, National Tsing Hua University, Hsinchu 30013, Taiwan, Republic of China

**Keywords:** Hybrid structure, ZnO nanorods, Graphene sheet, Transmittance, Hall measurement

## Abstract

A hybrid structure (HS) made of one-dimensional ZnO nanorods (NRs) and a two-dimensional synthesized graphene sheet was successfully constructed in this study. The uniform ZnO NRs were obtained by hydrothermal method and grown on a graphene surface that had been transferred to a polyethylene terephthalate substrate. The HS exhibited high transmittance (approximately 75%) over the visible wavelength range, even after cyclic bending with a small radius of curvature. Raman spectroscopy and Hall measurement were carried out to verify the chemical composition and electrical properties of the structure. Stable electrical conductance of the ZnO NR/graphene HS was achieved, and increase in carrier mobility decreased the resistance of the ZnO-with-graphene sheet in comparison with bare ZnO NRs.

## Background

Various investigations have concentrated on the development of promising materials with multifunctionality for emerging electronic and optoelectronic systems [1,2]. For example, interest has been growing in combining the specific properties of different dimensional structures on a flexible and transparent substrate. Recently, the composition of hybrid structures (HS) using one-dimensional (1D) and two-dimensional (2D) components to synthesize novel materials has been gaining much attention, especially for 1D ZnO nanorods (NRs) and 2D graphene sheets. Graphene, which consists of monolayers of *sp*^2^ hybrid carbon atoms, has been the most attractive carbon material in recent years [3-6]. Because of carbon-carbon covalent bonds, a graphene sheet exhibits extraordinary electrical and mechanical properties, including high intrinsic mobility (15,000~20,000 cm^2^/Vs) [7], a stretchable nature, and high thermal conductivity (approximately 5,300 W/mK) [8]. Moreover, with high optical transmittance and high chemical stability, graphene is a promising building block of window material for optoelectronic devices. In addition to graphene, transparent ZnO NRs with a wide bandgap are good candidates for use in next-generation electronics and optoelectronics [9-13]. Many methods have been developed to prepare ZnO NRs, including chemical vapor deposition (CVD) [14], vapor–liquid-solid epitaxy [15], and pulsed laser deposition [16]. However, these techniques are only applicable to limited substrate sizes and require high process temperatures, which are prohibitive for many practical applications. On the other hand, the hydrothermal process is regarded as a promising technique for the synthesis of ZnO NRs because it has several advantages, including the fact that it is a low-cost and low-temperature process that provides wafer-scale uniformity and high growth rates. Therefore, the hybridization of 2D graphene with 1D ZnO NRs has recently been reported for multifunctional applications, such as gas sensors [17], light-emitting diodes [18], solar cells [19], and piezoelectric nanogenerators [20]. In addition, ZnO is an excitingly attractive material for use as a transparent conducting oxide (TCO), but ZnO cannot solitarily exist as a TCO because of its intrinsic point defects [21,22]. To overcome this problem, increasing the carrier concentration or carrier mobility is effectively equivalent to decreasing the sheet resistance. In our opinion, ZnO NR/graphene HSs have characteristics that are of particular interest to the development of such structures for use as TCOs.

In this work, 1D ZnO NRs were synthesized by hydrothermal method onto a 2D graphene sheet to form an HS. High transmittance over the visible light region was obtained after synthesizing the ZnO NRs, and the sample displayed excellent mechanical properties after bending with a small radius. Notably, we essayed a Hall measurement of the HS, which consisted of ZnO NRs/graphene on a polyethylene terephthalate (PET) substrate.

## Methods

Each graphene sheet was prepared on a Cu foil by CVD and then spin-coated with a protective layer of poly(methyl methacrylate) (PMMA). The PMMA/graphene/Cu foil sample was immersed into an FeCl_3_/HCl solution for etching to strip the Cu foil. After etching, we retrieved the sample from the FeCl_3_/HCl solution, transferred it onto the PET substrate, and cleaned it with deionized water. Finally, the PMMA/graphene was soaked in an acetone solution to remove the PMMA and then washed with isopropyl alcohol [23]. A 100-nm ZnO seed layer was coated onto the graphene sheet with an E-gun evaporation system. Following this step, the ZnO NRs were grown in an equal molar aqueous solution of hexamethylenetetramine (HMTA) and zinc nitrate hexahydrate at 95°C for 2 h. The sample was cleaned with acetone and deionized water and then dried at room temperature. After the growth process, a morphological study of the ZnO nanostructures was performed with a JEOL JSM-6500 (Tokyo, Japan) field-emission scanning electron microscope (FE-SEM). Optical transmittance measurements were collected for nearly normal light incidence covering the spectral region from 400 to 800 nm with a standard UV-Visible spectrometer (ARN-733, JASCO, Easton, MD, USA). In this measurement, the noise level was approximately 0.002%. Raman spectrum was measured with a triple spectrometer (T64000, HORIBA Jobin Yvon SAS, Canal, France) equipped with a charge-coupled device cooled to 160 K. Hall measurement was performed with an Ecopia Hall effect measurement system (HMS-3000 ver 3.51.4).

## Results and discussion

To investigate the 3D hybrid nanostructure formed by combining 1D ZnO NRs with2D graphene, the ZnO seed layer was coated onto the graphene surface and annealed at a suitable temperature for the growth of ZnO NRs through hydrothermal method. The ZnO NRs presented here were obtained with a solution-based chemical synthesis. In a solution containing zinc nitrate hexahydrate and HMTA, hydroxyl ions were released through the thermal decomposition of the HMTA and reacted with zinc ions to form ZnO. The synthesis can be summarized in the following reactions:

(1) $$C_{6}H_{12}N_{4}+6H_{2}O↔6{\mathrm{CH}}_{2}O+4{\mathrm{NH}}_{3}$$

(2) $${\mathrm{NH}}_{3}+H_{2}O↔{\mathrm{NH}}_{4}^{+}+{\mathrm{OH}}^{-}$$

(3) $$2{\mathrm{OH}}^{-}+{\mathrm{Zn}}^{2+}\to {\mathrm{ZnO}}_{\left(s\right)}+H_{2}O$$

**Figure 1:**
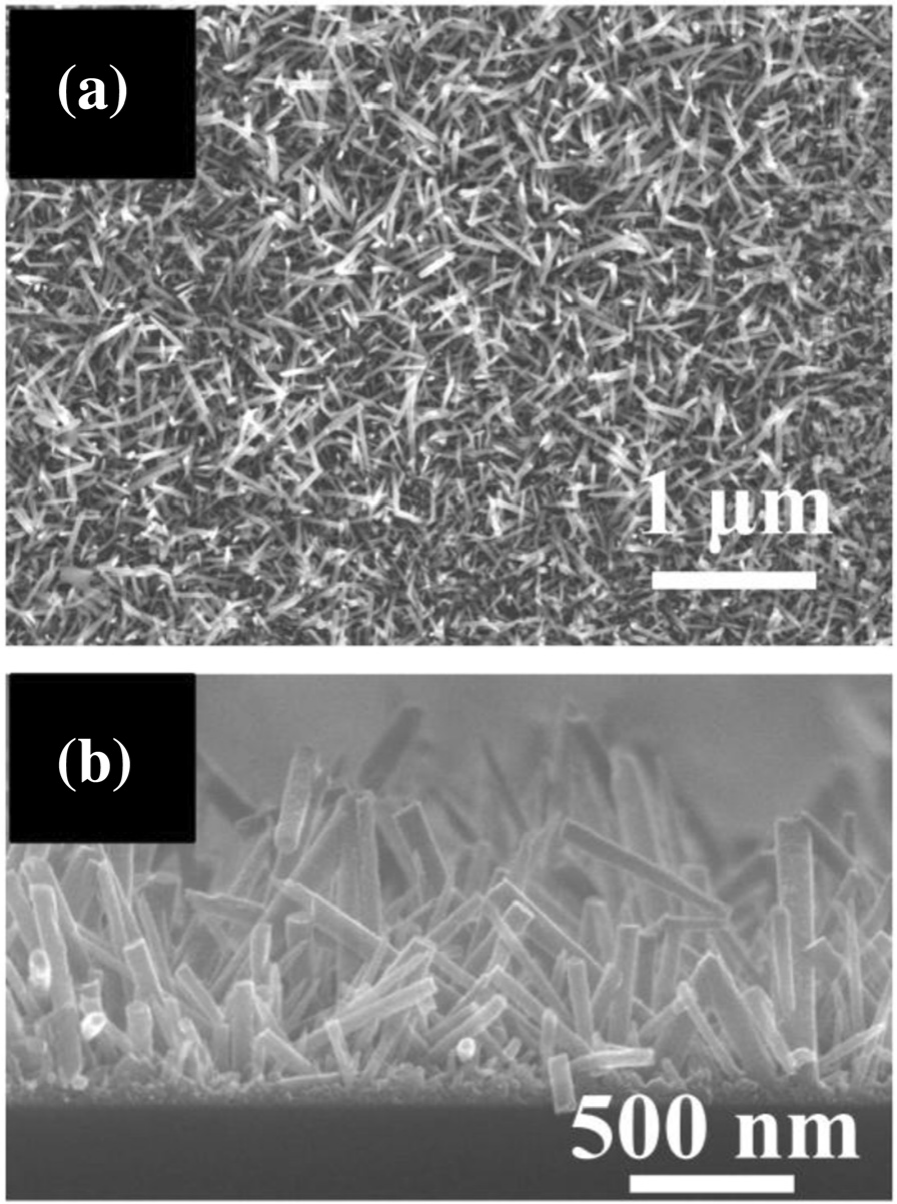
Plane-view (a) and cross-sectional (b) FE-SEM micrographs of ZnO NRs grown on graphene.

To observe the growth of the ZnO NRs on the graphene sheet, FE-SEM images were taken, as shown in Figure 1. Uniform ZnO NRs were successfully grown on the graphene surface. The average length and diameter of the NRs were 1 μm and 75 nm, respectively. The favored [0001] orientation of the ZnO NRs can be explained by the intrinsic high energy of the O^2−^ terminated surface, onto which the precursor molecules in the vicinity tend to be adsorbed [24]. Simultaneously, the HMTA supplies the solution with hydroxide ions, and Zn^2+^ cations usually form hydroxyl complexes as the precursors of ZnO.

**Figure 2:**
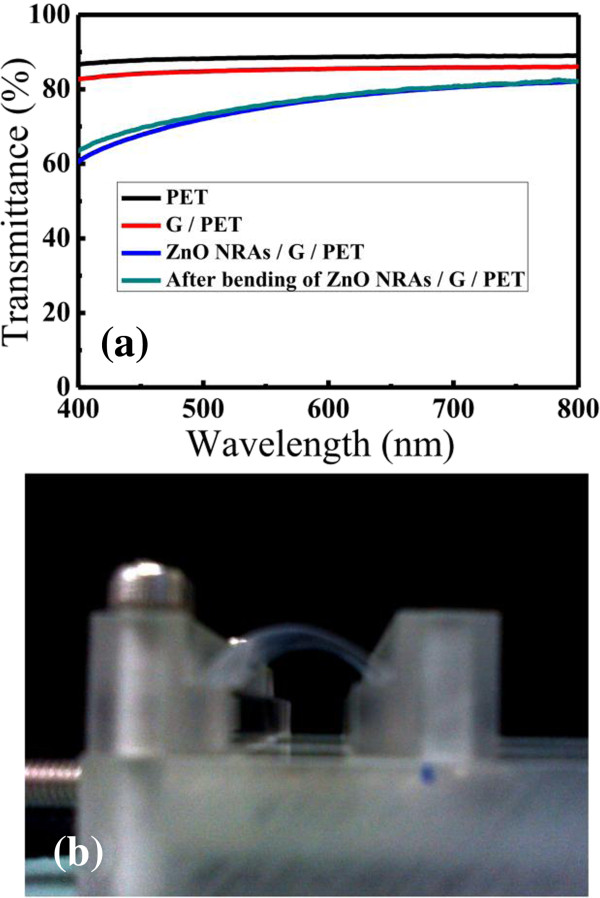
**Transmittance before and after bending and photographic images of ZnO NRs/graphene. (a)** Transmittance of bare PET, graphene/PET, and ZnO NRs/graphene/PET before and after bending. **(b)** Photographic images of flexible ZnO NRs/graphene on PET substrate in the bending state.

A concerning feature of the hybrid structure is that, although ZnO and graphene exhibit good optical transmittance in the visible spectral range, the scattering of light by ZnO NRs is suspected to lead to a decrease in transmittance to a certain extent. The optical transmittance of the ZnO NR/graphene hybrid structure was estimated by fabricating the structures on PET substrates. Figure 2a shows the optical transparency of PET, graphene/PET, and ZnO NRs/graphene/PET before and after bending. The average transmittance measured by UV–vis spectroscopy over the visible wavelength range of 400 to 800 nm was greater than 80% before ZnO NR growth, which decreased to 75% after NR growth. This slight decrease in the transmittance is attributed to absorption by ZnO NRs, which have a wide bandgap (3.37 eV). Even when ZnO NRs were grown on graphene, the sample still maintained high transmittance. One of the attractive features of graphene is its outstanding mechanical strength and elasticity [25]. To establish a stable performance of the hybrid structure after bending, the ZnO NRs/graphene on a PET substrate was bent with an approximately 0.7-mm radius 120 times (Figure 2b). No serious mechanical failure was evident in our samples. The high optical transmittance remained near 75%; in fact, it was even slightly higher than before the bending.

**Figure 3:**
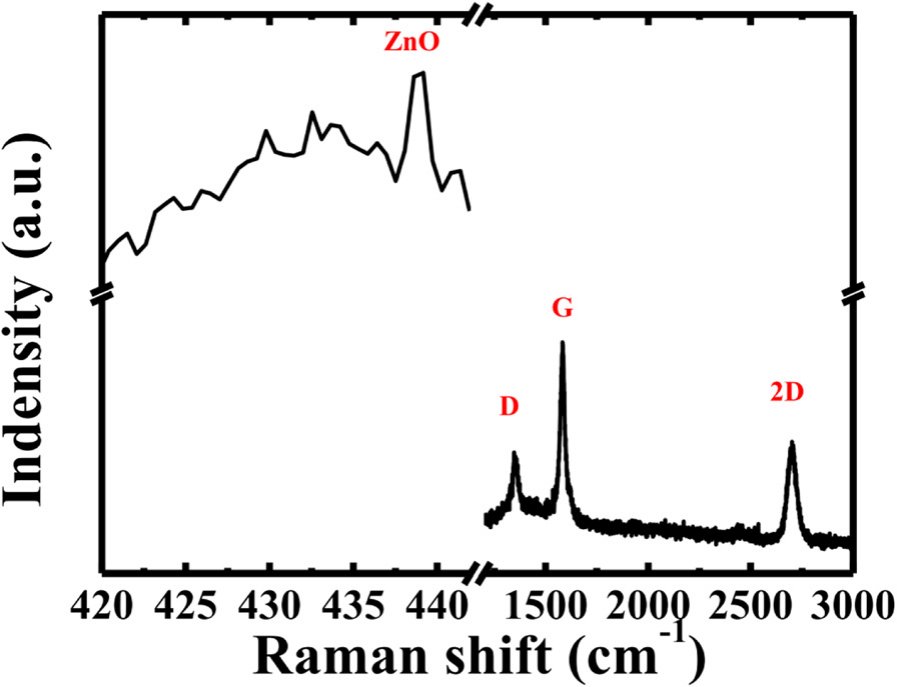
Raman spectrum of the ZnO NRs/graphene sheet.

Raman spectroscopy is a promising method for inspecting the ordered/disordered crystal structures of carbonaceous materials and the different layer characteristics of graphene. It was also used to prove that ZnO nanostructures were grown on graphene surface. The usual peak at 437 to 439 cm^−1^ corresponds to the *E*_2_ mode of the ZnO hexagonal wurtzite structure [26]. The G peak at approximately 1,580 cm^−1^ is attributed to the *E*_2g_ phonon of C *sp*^2^ atoms, and the D peak at approximately 1,350 cm^−1^ is accredited to local defects and disorder, such as the edges of graphene and graphite platelets [27,28]. Moreover, a 2D peak at approximately 2,700 cm^−1^ has also been found that may be related to the formation and number of layers of the graphene [29]. Figure 3 shows that the Raman spectrum of the ZnO/graphene exhibits the ZnO peak at 439 cm^−1^, the D peak at 1,353 cm^−1^, the G peak at 1,586 cm^−1^, and the 2D peak at 2,690 cm^−1^. The formation of few-layer graphene was verified by the intensity ratio of the G peak to that of the 2D peak, which was approximately 1 to 1.5, and by the position of the 2D peak [30,31]. In a word, the characteristics of ZnO and graphene were confirmed by the Raman spectrum.

**Figure 4:**
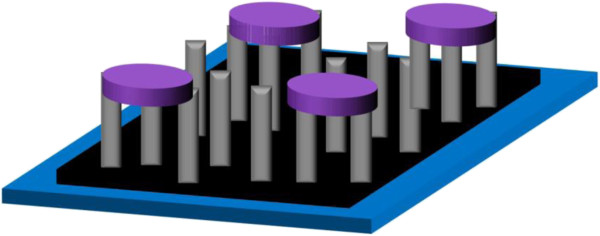
A schematic illustration of the device fabricated for Hall measurement.

The device structure was fabricated as shown in Figure 4 for Hall measurement. Four electrodes of 200 nm in thickness (100-nm Ti and 100-nm Ag) were located on the four terminals of the ZnO NR layer that was grown on the graphene surface. The electrical conductivity of the ZnO NRs/graphene was obtained and is presented in Table 1. The ZnO film exhibited a high sheet resistance and low charge-carrier mobility before being combined with the graphene sheet. It has previously been discovered that the application of high-mobility graphene is a promising method of addressing this issue of high sheet resistance and low charge-carrier mobility [32]. Therefore, the Hall measurement of the novel hybrid structure, ZnO NRs/graphene on PET, was carried out. The charge-carrier mobility was as high as 124.8 cm^2^/Vs, 18 times higher than that of the ZnO film. It has been reported that there is an important relationship between mobility and sheet resistance because the carriers can be easily scattered by lattice defects [33]. Accordingly, an enhancement of the mobility would decrease the sheet resistance and thereby promote the electrical conductivity. As a result, a low sheet resistance can be attained because the introduction of a graphene sheet leads to an increase in the overall mobility. Similarly, the stationary electrical performance after bending was an issue of concern. From Table 1, it can be seen that high mobility and low sheet resistance were still observed after bending for 120 repetitions. The hybrid structure of ZnO NRs/graphene has not yet been fully optimized for use as a TCO layer. However, we have demonstrated its great potential for application in optoelectronic devices.

**Table 1:** The results of Hall measurements of ZnO and ZnO NRs/graphene on PET substrate

	**Rs**	**Carrier concentration**	**Mobility**
	**(Ω cm)**	**(cm^3^)**	**(cm^2^/Vs)**
ZnO	0.9948	10^12^	6.72
ZnO NRs/graphene	0.2416	10^12^	124.8
ZnO NRs/graphene after bending	0.2426	10^12^	120.6

## Conclusions

Uniform ZnO NRs were obtained by hydrothermal method and grown on a graphene surface that had been transferred to a PET substrate. The ZnO NR/graphene HS exhibited high transmittance (approximately 75%) over the visible wavelength range, even after cyclic bending with a small radius of curvature. Stable electrical conductance of the ZnO NR/graphene HS was achieved, and the improvement of the ZnO sheet resistance by the incorporation of the graphene sheet can be attributed to the resultant increase in carrier mobility.

## Competing interests

The authors declare that they have no competing interests.

## Authors' contributions

RJC was the principal investigator and is also the corresponding author of this paper. ZCL and PKY were in charge of material preparation and characterization. KYL contributed to data analysis. SFJ and PWC contributed to graphene synthesis. All authors collaborated to complete this research and to compile this manuscript. All authors read and approved the final manuscript.
